# PA/MR imaging-guided precision phototherapy and efficacy evaluation of hepatocellular carcinoma utilizing a targeted multifunctional nanoprobe

**DOI:** 10.3389/fimmu.2025.1605048

**Published:** 2025-06-11

**Authors:** Shuo Qi, Lei Zhou, Weinan Liu, Lian Shen, Yuping Yao, Bingzhang Tian, Changjun Liu, Wei Cheng, Kang Chen

**Affiliations:** Department of Hepatobiliary Surgery, Hunan Provincial People’s Hospital, The First Affiliated Hospital of Hunan Normal University, Changsha, Hunan, China

**Keywords:** photoacoustic (PA) imaging, magnetic resonance (MR) imaging, photothermal therapy (PTT), photodynamic therapy (PDT), hepatocellular carcinoma

## Abstract

**Introduction:**

Early-stage hepatocellular carcinoma (HCC) poses a significant challenge due to its poor prognosis, necessitating advancements in diagnostic and therapeutic strategies. The integration of near-infrared photoacoustic (PA) imaging with magnetic resonance (MR) imaging offers enhanced temporal and spatial resolution, exceptional optical contrast, and profound tissue penetration, positioning this combination as a highly promising technique for accurate and sensitive HCC diagnosis.

**Methods:**

In this study, we developed a multifunctional and highly biocompatible nanoplatform, designated as ICG/Mn-PDA-PEG-CXCR4 (IMPP-c). This nanoplatform is designed to diagnose and treat early-stage HCC through PA/MR imaging-guided noninvasive photothermal therapy (PTT) and photodynamic therapy (PDT).

**Results:**

Both *in vitro* and *in vivo* experiments demonstrated enhanced accumulation of IMPP-c nanoparticles (NPs) within HCC. Notably, the dual-modal PA/MR imaging facilitated by IMPP-c achieved high resolution and substantial deep tissue penetration, enabling precise localization of early orthotopic small hepatocellular carcinoma (SHCC) lesions. *In vivo* tumor phototherapy experiments, guided by PA/MR imaging, revealed that SHCC was completely eradicated through noninvasive PTT/PDT without recurrence. Additionally, the metabolism of IMPP-c NPs was observed in major organs throughout the treatment process, confirming its reliable biocompatibility.

**Discussion:**

This study introduces a novel method for diagnosing and implementing non-invasive therapeutic interventions in early HCC using nanoparticle systems such as IMPP-c, paving the way for potential future clinical applications.

## 1 Introduction

Hepatocellular carcinoma (HCC) is a leading cause of cancer-related mortality worldwide, characterized by a 5-year survival rate that falls below 18% (1, 2). This underscores its status as one of the deadliest forms of cancer globally (3). The lack of early diagnostic methods and effective treatments often leads to patients missing curative interventions due to late diagnosis (4, 5). In clinical practice, the early detection of small hepatocellular carcinoma (SHCC) presents significant challenges, primarily due to low sensitivity and inadequate penetration depth (6, 7). Ultrasound (US) imaging and X-ray computed tomography (CT) are commonly employed in clinical settings to visualize solid tumors located within deep tissues (8, 9). However, these modalities exhibit limitations when it comes to identifying SHCC lesions smaller than a few millimeters (mm). Liver-specific enhanced magnetic resonance (MR) imaging is increasingly being recognized in clinical practice because it can accurately detect SHCC lesions approximately 10-mm in size and identify satellite lesions within the liver, but its imaging speed, price, etc. limit its clinical application (10, 11). Therefore, clinically, a novel imaging technology is needed to precisely image SHCC, enabling early detection.

Photoacoustic (PA) imaging has garnered substantial attention due to its exceptional biocompatibility, sensitivity, and molecular imaging capabilities, which facilitate precise SHCC diagnosis (12, 13). PA imaging circumvents the limitations of traditional ultrasound and optical imaging by leveraging the low scattering of ultrasound in tissues, coupled with the high sensitivity and resolution of optical techniques (14, 15). PA imaging technology can further enhance precision and sensitivity through the use of PA contrast agents, analogous to those used in enhanced MRI, providing more accurate delineation of tumor boundaries and diagnostic information (16, 17). Consequently, the innovative integration of PA imaging with MRI may offer a novel approach for the early diagnosis of SHCC.

PA molecular imaging is a significant technique within the realm of molecular imaging, and it has found extensive applications in the biomedical field. PA imaging contrast agents serve as crucial media for facilitating PA molecular imaging (18, 19). The reported PA contrast agents primarily encompass various dyes, semiconductor materials, and metal nanoparticles, such as manganese (Mn), gold, and platinum, all of which exhibit exceptional light absorption capabilities and are increasingly employed to enhance PA imaging (20–22). Upon light absorption, thermal expansion can be induced for PA imaging; concurrently, photothermal and photodynamic effects may also be generated to enable photothermal therapy (PTT) and photodynamic therapy (PDT) (23–25). Mn-based contrast agents possess distinctive advantages in augmenting MRI of the liver and nervous system. Furthermore, their combination with Mn-derived polydopamine (PDA) nanocarriers demonstrates promising performance in both PA imaging and PTT, along with favorable biocompatibility (26, 27). Indocyanine green (ICG) stands out as the most widely utilized near-infrared fluorescent dye and photosensitizer; it is extensively applied in clinical surgical navigation and has received approval from the U.S. Food and Drug Administration (FDA) (28, 29). Consequently, the PDA complex combined with ICG proves suitable for tumor detection and treatment through modalities such as PA imaging, PTT/PDT for tumors, as well as MRI applications.

In this work, we developed a highly biocompatible and multifunctional nanoplatform for the early theranostic management of subcutaneous/orthotopic HCC through PA/MR bimodal imaging-guided PTT/PDT. By encapsulating Mn^2+^ and ICG within PDA nanoparticles, we synthesized a blackbody-like agent, designated as ICG/Mn-PDA. To enhance targeting specificity, we modified the surface of ICG/Mn-PDA with sulfhydryl-terminated polyethylene glycol (SH-PEG) and conjugated it with an antibody targeting CXCR4, a highly expressed HCC marker, resulting in the formation of actively targeting nanoparticles (IMPP-c). IMPP-c exhibited an exceptional photothermal conversion efficiency (PCE) of 67.9% under 808-nm laser excitation, endowing the efficient photothermal therapy effect to both subcutaneous and orthotopic HCC. Both *in vitro* and *in vivo* experiments demonstrated the targeted enrichment of IMPP-c within HepG2 cells and HCC. The PA/MR dual-modality imaging facilitated by IMPP-c revealed deep tissue penetration and high resolution, enabling precise localization of early-stage orthotopic SHCC lesions. *In vivo* PTT/PDT experiments, guided by PA/MR imaging, indicated that SHCC could be completely eradicated noninvasively without recurrence. Furthermore, IMPP-c metabolism was observed within 48 hours across major organs, confirming its reliable biocompatibility. Our findings represent a significant advancement in the diagnosis and noninvasive therapeutic intervention for early-stage HCC using nanoparticle-based systems like IMPP-c, paving the way for potential clinical translation.

## 2 Materials and methods

### 2.1 Chemicals

All chemical reagents necessary for the synthesis of IMPP-c NPs were procured from commercial sources and were utilized without any further purification. Manganese (II) chloride (MnCl_2_), Dopamine-hydrocholoride (DA-HCl), and ICG were supplied by Sigma-Aldrich (Shanghai, China). D-Luciferin and PEG-SH (5 kDa) were procured from Aladdin (Shanghai, China). Cell culture medium, PBS, trypsin, and the cell counting kit-8 (CCK-8) were obtained from Thermo Fisher Scientific (Shanghai, China). The anti-CXCR4 antibody (ab208128) was acquired from Abcam (Shanghai, China). A detailed account of the synthesis process for IMPP-c NPs is provided in the Supporting information.

### 2.2 Characterization

The morphology was analyzed using a transmission electron microscope (TEM) (TF20, JEOL, Japan). The Zeta potential and hydrodynamic particle size were measured with a Zetasizer Nano ZS (SZ-100, Horiba, Japan). The UV-Vis-NIR absorption spectrum of IMPP-c NPs was recorded utilizing a Shimadzu spectrophotometer (Sintec Optronics Technology, Singapore). Fluorescent spectra were obtained using a spectrofluorometer (Hitachi, Japan). Temperature variations were monitored with a thermal camera (FLIR One Pro, USA). Serum stability was assessed by dissolving the sample (0.2 mg·mL^−1^) in serum to simulate physiological conditions at 37°C for durations of 6, 12, 24, and 48 hours *via* the Zetasizer Nano ZS.

### 2.3 *In vitro* PA and MR imaging

Different concentrations of IMPP-c aqueous solutions (0.025, 0.05, 0.1, 0.2, and 0.4 mg·mL^-1^) were sealed in distinct rubber capillaries. Subsequently, the PA signals of each sample at a wavelength of 808 nm were detected and quantified using the PA imaging system (Vevo LAZR-X, Visual Sonics, Fujifilm, Japan), which is equipped with an LZ-550 linear sensor operating at a center frequency of 40 MHz. Concurrently, the PA stability of IMPP-c NPs was assessed utilizing the PA imaging system following laser irradiation for durations ranging from 0 to 30 minutes. MRI was conducted using a 9.4 T BioSpec 94/30 (Bruker, Germany) equipped with an animal abdominal coil. Various concentrations of IMPP-c aqueous solutions (0.025, 0.05, 0.1, 0.2, and 0.4 mg·mL^-1^) were prepared in EP tubes and positioned on a wax holder to assess the T_1_-weighted signals. The T_1_-weighted signals were acquired by manually delineating the region of interest (ROI) on the images, and the relaxation rate (r_1_) of IMPP-c aqueous solutions was determined as the reciprocal of T_1_ (i.e., r_1_ = 1/T_1_).

### 2.4 *In vitro* PTT and PDT

Different concentrations of IMPP-c aqueous solutions, ranging from 0 to 0.4 mg·mL^−1^, were injected into quartz cuvettes. Temperature changes in these samples were recorded and photographed every two minutes using a FLIR camera, under an 808-nm laser emitting at a power output of 0.8 W·cm². The laser was cycled on and off five times to allow the temperature to equilibrate during heating and subsequently cool naturally to room temperature, thereby assessing the photothermal stability of IMPP-c NPs. To verify the photo-switchable PDT potential of IMPP-c NPs, we utilized a single-line oxygen sensor green (SOSG) as a probe for detecting singlet oxygen (¹O₂). The variation in SOSG fluorescence intensity, assessed both under laser irradiation and in its absence, indicates the levels of ¹O₂ present across each experimental group. Additionally, ethanol was combined with IMPP-c, ICG, and PBS followed by the introduction of 1,3-diphenylisobenzofuran (DPBF). The depletion of DPBF after approximately five minutes of exposure to an 808-nm laser serves as an indicator for reactive oxygen species (ROS) generation and reflects the efficacy of PDT performance.

### 2.5 Cellular targeting uptake of IMPP-c NPs

The human HCC cell lines HepG2 and HCCLM3, and the normal human hepatocyte LO2 cells (American Type Culture Collection, ATCC) were cultured in 89% Dulbecco’s Modified Eagle Medium (DMEM), supplemented with 10% fetal bovine serum (FBS) and 1% penicillin-streptomycin. The cultures were maintained at a temperature of 37°C in a humidified atmosphere containing 5% CO_2_. For confocal imaging, HepG2 and LO2 cells were seeded into different laser confocal dish and allowed to incubate for 24 hours. Following this incubation period, the culture medium containing IMPP-c NPs (0.4 mg·mL^-1^) was added to each dish and further incubated for an additional 4 hours. Subsequent to these treatments, the NPs-containing culture medium was removed, and the cells were fixed using 4% paraformaldehyde (500 µL for 10 minutes). The cell nuclei were then stained with DAPI at a concentration of 1.5 µg·mL^-1^ for 12 minutes before rinsing the dishes three times with PBS (1 mL each time, over a period of 10 minutes). Finally, fluorescence images of the cells were acquired utilizing a laser confocal microscope (Leica, Germany). For TEM analysis of HepG2 cells, the cells underwent incubation with IMPP-c NPs for a duration of 4 hours before being prepared on copper grids suitable for TEM observation.

### 2.6 Cellular dark and photo toxicity

To evaluate the toxicity of IMPP-c NPs under dark conditions, a CCK8 assay was performed. HepG2, HCCLM3 and LO2 cells were seeded at appropriate densities into separate 96-well plates and incubated for 24 hours. Subsequently, the growth medium was replaced with a mixture containing varying concentrations of NPs (0, 0.05, 0.1, 0.2, and 0.4 mg·mL^-1^) and incubated in darkness for an additional 24 hours. Following this incubation period, the medium-NPs mixture was removed; CCK solution was then added to each well for further incubation lasting another 4 hours. The absorbance at a wavelength of 450 nm in each well was measured using a microplate reader. To assess the cellular PTT and PDT efficacy of IMPP-c NPs, HepG2 and HCCLM3 cells were also plated in individual wells within a separate 96-well plate and cultured for an initial duration of 24 hours. Afterward, the culture medium was exchanged with one containing various concentrations of IMPP-c NPs (0, 0.05, 0.1, 0.2, and 0.4 mg·mL^-1^), followed by incubation for an additional period of 4 hours prior to exposure to an infrared laser at a wavelength of 808-nm for 10 minutes; subsequently allowing another incubation phase in darkness that lasted an additional 24 hours thereafter. The cell survival rates across all samples were determined through the aforementioned CCK8 assay methodology; final results represent averages obtained from three independent experimental repetitions. To accurately reflect cell survival status, HepG2 and HCCLM3 cells were independently cultured in separate dishes for 24 hours before replacing the culture medium with one containing IMPP-c NPs while continuing incubation for an additional 4 hours period post-replacement. Following this treatment step; samples from both Laser + IMPP-c/PBS group underwent exposure to an infrared laser at wavelengths of 808-nm over 10 minutes while those designated as IMPP-c/PBS remained unirradiated during this interval. Subsequently these samples underwent staining utilizing Calcein-AM as well as propidium iodide (PI) over 15 minutes’ time span after which cell fluorescence images were acquired *via* fluorescence microscopy.

### 2.7 *In vivo* and *ex vivo* PA imaging

*In vivo* PA imaging was conducted using subcutaneous/orthotopic tumor-bearing BALB/c nude mice. Following anesthesia, IMPP-c NPs (30 mg·Kg^-1^)/ICG were administered to the tumor-bearing mice *via* the tail vein. To assess the accumulation time of IMPP-c NPs in both subcutaneous and orthotopic tumor regions, real-time monitoring of the PA signal within the tumor area was performed utilizing a PA imaging system over a subsequent 24-hour period. Furthermore, to further verify the accumulation of IMPP-c NPs in the orthotopic tumors, surgical procedures were carried out on the mice at designated time points. The tumors along with major organs—including heart, liver, spleen, lung, and kidney—were excised for analysis. Subsequently, these excised tumors and organs were imaged using the PA imaging system.

### 2.8 *In vivo* MR imaging

The BALB/c nude mice bearing subcutaneous/orthotopic tumors were utilized for *in vivo* MR imaging studies. To investigate the distribution of the NPs at the tumor site, IMPP-c NPs (administered at a dose of 30 mg·Kg^-1^)/ICG were injected *via* the tail vein into mice with subcutaneous/orthotopic tumors. The mice were anesthetized using 2% isoflurane and connected to an ECG device for continuous monitoring of electrocardiogram, respiration, and body temperature. Tumors located in both subcutaneous and orthotopic mice were imaged utilizing the Turbo RARE T_1_ imaging sequence (field of view: 3 cm; slice thickness: 0.5 mm).

### 2.9 *In vivo* PTT/PDT

The *in vivo* PTT and PDT experiment was performed using HepG2 tumors implanted in BALB/c nude mice, encompassing both subcutaneous and orthotopic tumor models. Prior to the initiation of the experiment, subcutaneous tumors exhibited an approximate volume of 80 mm³, while all orthotopic SHCC formations were confirmed *via* an *in vivo* bioluminescence imaging system. Subsequently, the mice were categorized into four groups: treatment groups (laser + IMPP-c/PBS group) and a control group (IMPP-c/PBS group). In the laser + IMPP-c/IMPP-c group, following intravenous injection at a dosage of 30 mg·kg^−1^, IMPP-c NPs were administered; conversely, an equivalent volume of PBS was injected for the laser + PBS/PBS control group. Tumor locations were verified by gross examination for subcutaneous tumors and through optical imaging guidance employing bioluminescence imaging for orthotopic tumors. At a predetermined accumulation time point for IMPP-c NPs, an 808-nm laser with an intensity of 0.8 W·cm⁻² was utilized to irradiate the tumor sites in mice assigned to the laser treatment group over a duration of 12 minutes—this method being minimally invasive without requiring laparotomy—while no irradiation occurred in either control group. During PTT/PDT treatment sessions, temperature changes within the tumor area were monitored using infrared thermal imaging. Following this procedure, tumor volumes within the subcutaneous treatment cohort were recorded every other day; meanwhile, measurements for orthotopic tumors were taken on days 7 and 14 post-treatment. Bioluminescence imaging as well as MR imaging assessments were performed on each mouse cohort using an *in vivo* bioluminescence/MR imaging system to quantify results accurately. All mice underwent evaluations regarding their general condition and weight every other day throughout this period. On day 15 post-treatment completion, euthanasia was executed through cervical dislocation across all experimental groups. Subsequently, the treated tumors and major organs were surgically excised for comprehensive histological analysis utilizing hematoxylin and eosin (H&E) staining techniques.

### 2.10 Statistical analysis

The data are presented as mean ± standard deviation (M ± SD), and statistical comparisons were conducted using the Analysis of ANOVA with *post-hoc* corrections as necessary. Statistical analysis and figure plotting were performed utilizing MATLAB software (MathWorks, USA) and Origin software (OriginLab, USA). *P* value of less than 0.05 was considered statistically significant.

## 3 Results and discussion

### 3.1 Characterization of IMPP-c NPs

**Figure 1:**
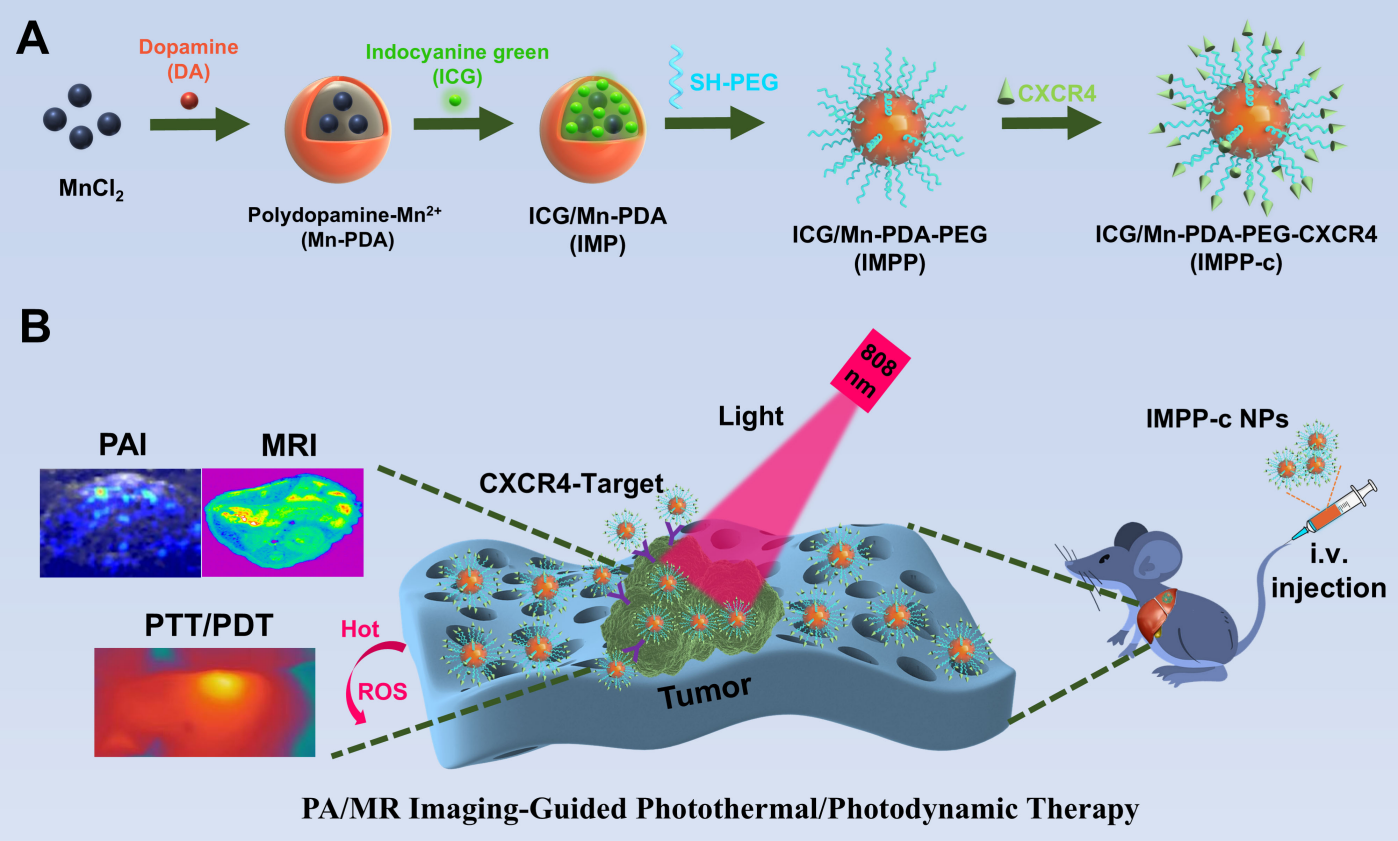
Flow chart of the experiment. **(A)** The development of a multifunctional theranostic nanoplatform (IMPP-c). **(B)** Applications of IMPP-c NPs in PA/MR imaging-guided photothermal and photodynamic therapy for HCC-bearing nude mice.

**Figure 2:**
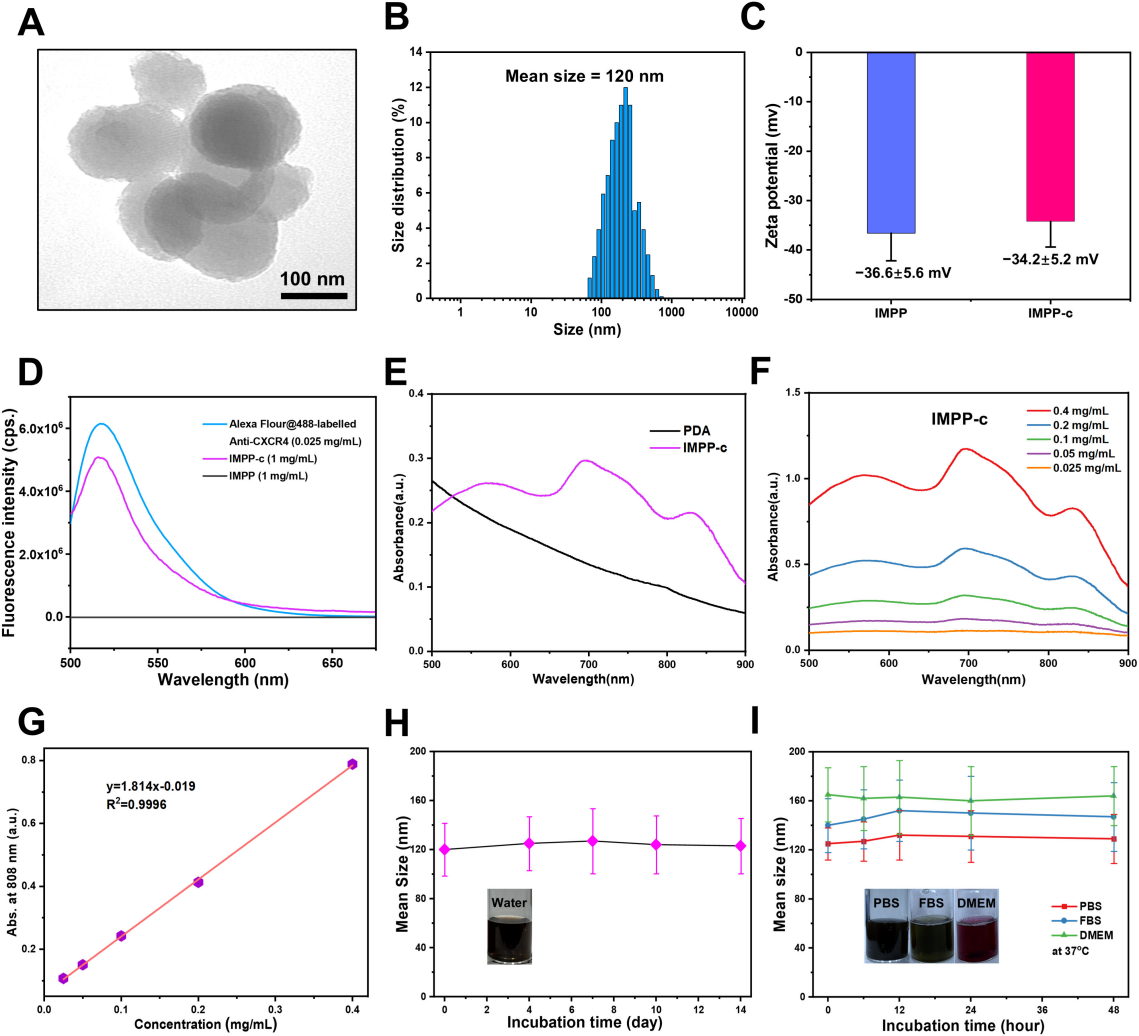
Characterization of IMPP-c NPs. **(A)** TEM images of IMPP-c NPs. **(B)** DLS size distribution of IMPP-c NPs, with a mean size of 120 nm. **(C)** Zeta potential measurements for both IMPP NPs and IMPP-c NPs. **(D)** Fluorescent spectra obtained from Alex-Fluor-labeled Anti-CXCR4, along with those from both IMPP-c and IMPP; the excitation wavelength used was 470 nm. **(E)** UV-Vis-NIR spectra of PDA and the aqueous solution of IMPP-c. **(F)** UV-Vis-NIR spectra of aqueous dispersions of IMPP-c at varying concentrations: 0.025, 0.05, 0.1, 0.2, and 0.4 mg mL^−1^. **(G)** Absorbance plot according to Lambert–Beer law for absorption at a wavelength of 808 nm. **(H)** Size stability assessment of IMPP-c in deionized water. **(I)** Stability evaluation via DLS for IMPP-c NPs during incubation in various media—PBS, FBS, and DMEM—at 37°C to simulate physiological conditions.

As depicted in **Figure 1**, the reaction process can be optimized through the incorporation of ICG/Mn precursors and anti-CXCR4 on the surface. This approach facilitates the development of multifunctional targeted PDA NPs, designated as ICG/Mn@PDA-PEG-CXCR4 (abbreviated as IMPP-c), which are intended for PA/MR imaging and targeted phototherapy applications. TEM imaging reveals that the particle size of IMPP-c is approximately 100-120 nm (as shown in **Figure 2A**), which aligns closely with the particle size measured *via* DLS testing (120 ± 14.5 nm) (as presented in **Figure 2B**). The zeta potentials of both IMPP and IMPP-c were recorded at -36.6 ± 5.6 mV and -34.2 ± 5.2 mV, respectively. This variation in zeta potential indicates that anti-CXCR4 has been electrostatically adsorbed onto the surface of IMPP NPs, as illustrated in **Figure 2C**. Furthermore, discrepancies observed in fluorescence spectra between IMPP and IMPP-c provide additional evidence supporting successful attachment of anti-CXCR4 through electrostatic adsorption, with an estimated antibody loading amount calculated to be approximately 1.6% based on fluorescence peak analysis (as indicated in **Figure 2D**). Meanwhile, the study also confirmed the quenching of ICG fluorescence after the synthesis of IMPP NPs. The UV-NIR spectra for both IMPP-c and PDA NPs reveal distinct NIR absorption peaks at wavelengths of 690 nm and 830 nm for IMPP-c (**Figure 2E**), while a significant decrease in absorption is noted for PDA with increasing wavelength. As illustrated in **Figures 2F** and **2G**, an increase in concentration correlates with a gradual rise in NIR absorption for IMPP-c, demonstrating a linear relationship at an 808-nm wavelength characterized by R² =0.9996. As illustrated in **Figure 2H**, IMPP-c NPs exhibit excellent dispersion in deionized (DI) water, and their particle size remains relatively stable after being stored at 4°C for a duration of 14 days. Furthermore, as demonstrated in **Figure 2I**, IMPP-c NPs maintain good dispersion across various liquids—including phosphate-buffered saline (PBS), fetal bovine serum (FBS), and Dulbecco’s Modified Eagle Medium (DMEM)—with no significant changes in particle size observed following storage at 37°C for 48 hours, thereby simulating physiological conditions. In summary, these findings indicate that IMPP-c possesses an appropriate particle size along with favorable NIR absorption characteristics while exhibiting commendable stability within biological fluids; thus, positioning it well for potential biomedical applications.

### 3.2 PA/MR imaging and PTT/PDT properties *in vitro*

**Figure 3:**
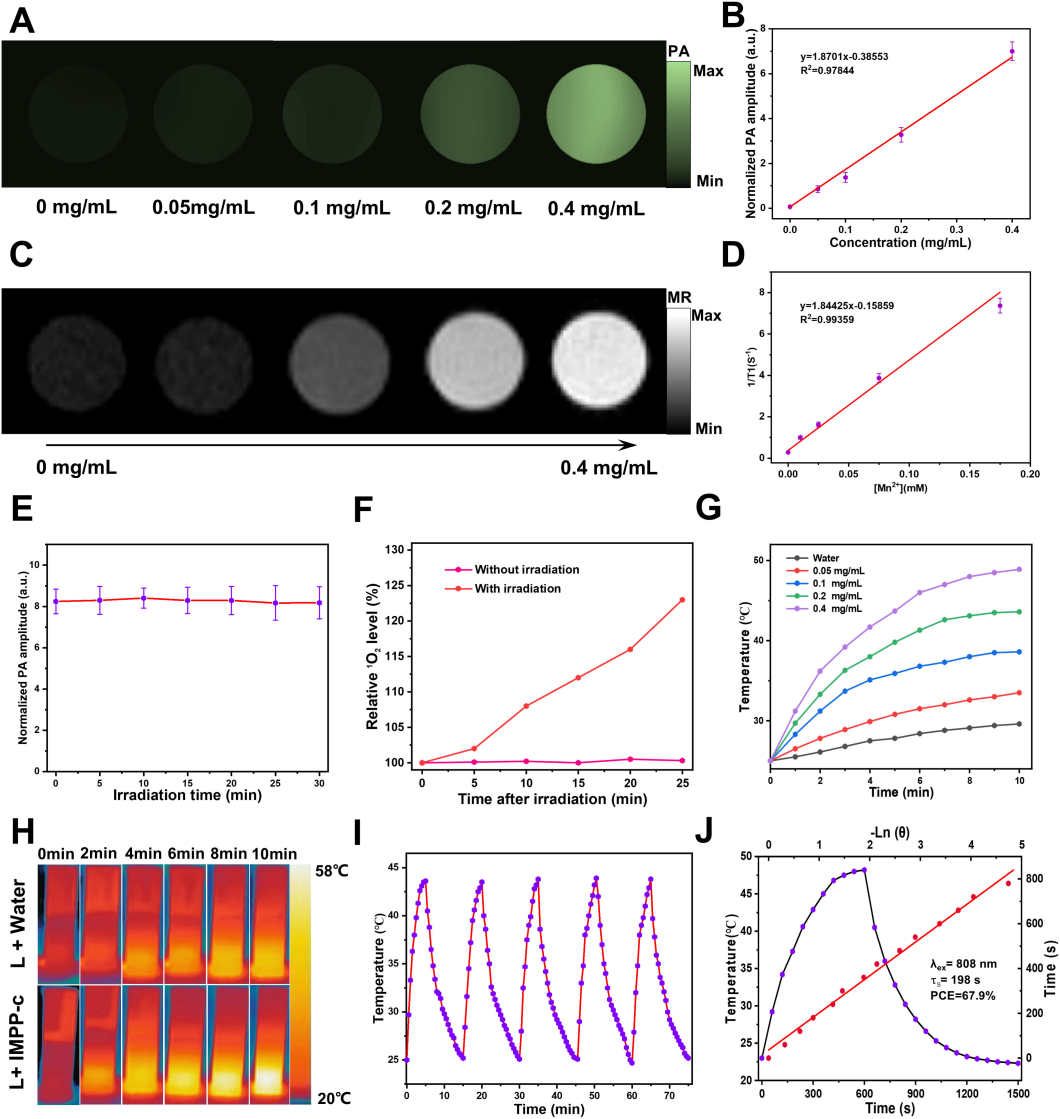
PA/MR signals and photothermal properties *in vitro*. **(A)** Concentration-dependent PA images of IMPP-c dispersions at a wavelength of 808 nm. **(B)** The corresponding linear regression curve illustrating the relationship between PA intensity and concentrations of IMPP-c. **(C)** MR images of IMPP-c dispersions at varying concentrations. **(D)** Relaxation rates 1/T1 (s^-1^) of IMPP-c as a function of Mn^2+^ concentrations (mM). **(E)** Stability test for PA signals from IMPP-c NPs under continuous 808-nm laser irradiation over a duration of 30 minutes. **(F)** Comparison of relative singlet oxygen (^1^O_2_) levels detected by SOSG in groups with and without laser irradiation. **(G)** Temperature elevation curves for IMPP-c NPs across different concentrations (0 - 0.4 mg mL^−1^) during exposure to laser irradiation at 808 nm with an intensity of 0.8 W·cm⁻². **(H)** Infrared thermal images depicting IMPP-c at a concentration of 0.4 mg mL^−1^ and deionized water in quartz cuvettes after being subjected to 10 minutes of 808-nm laser irradiation. **(I)** Photothermal heating and cooling cycles observed for IMPP-c NPs upon exposure to an 808-nm laser source. **(J)** Comprehensive heating and cooling profiles along with linear regression analysis for determining the time constant τs.

IMPP-c NPs exhibit strong NIR absorption capabilities, rendering them suitable for NIR PA imaging. The PA imaging system was employed to visualize IMPP-c NPs *in vitro*. As illustrated in **Figures 3A** and **3B**, at a wavelength of 808-nm and a light intensity of 5.0 mJ·cm⁻², the PA intensity demonstrated a positive correlation with the concentration of IMPP-c NPs, which ranged from 0 to 0.4 mg·mL^−1^ (R² = 0.97844). Subsequent stability tests indicated that the IMPP-c NPs maintained reliable optical stability after irradiation with an 808-nm laser for a duration of 30 minutes (20 pulses/second at a light intensity of 5 mJ·cm⁻²), as shown in **Figure 3E**; no significant changes were detected in the PA signal by the PA imaging system. Furthermore, Mn²⁺ incorporated within the IMPP-c NPs contributes to a robust magnetic resonance (MR) imaging signal. **Figures 3C** and **3D** present MR images along with quantitative analyses of IMPP-c dispersions at varying concentrations. The relaxation rates 1/T1 (s^-1^) is directly proportional to Mn concentration (mM) (R² = 0.99359). The strong PA/MR signals combined with excellent optical stability indicate that IMPP-c serves as an outstanding contrast agent for *in vivo* biological imaging.

Moreover, we evaluated the NIR PTT and PDT performance of IMPP-c NPs prior to *in vivo* application. The efficacy of PDT with IMPP-c NPs was validated using the SOSG ^1^O2 detection probe, which exhibited significant generation of reactive oxygen species (ROS), thereby indicating remarkable effectiveness in PDT (see **Figure 3F**). As depicted in **Figures 3G** and **3H**, the temperature of the IMPP-c NPs solution at varying concentrations (ranging from 0 to 0.4 mg·mL^−1^) increased to differing extents under laser irradiation. After 10 minutes of exposure to an 808-nm laser, the temperature for the group at a concentration of 0.4 mg·mL^−1^ elevated from 23°C to 49.2°C when subjected to an energy density of 0.8 W·cm⁻²; in contrast, deionized water exposed to similar laser conditions only experienced a temperature rise of 5.8°C. Furthermore, we assessed the PTT stability of IMPP-c NPs through 5 cycles of on/off laser testing (refer to **Figure 3I**), complemented by morphological examinations *via* TEM as shown in **Supplementary Figure S1** in **Supporting Information**; no significant changes in temperature or morphology were observed, confirming exceptional photothermal stability. As shown in **Figure 3G**, the photothermal conversion efficiency (PCE) for IMPP-c NPs was calculated through linear regression analysis against both IMPP-c and DI water values, yielding a PCE value of 67.9%—a result derived from methodologies detailed within the **Supporting information**—which ranks among the highest for both organic and inorganic materials as indicated in **Table 1 in the Supporting Information** The pronounced photothermal conversion characteristics exhibited by IMPP-c may be attributed to non-radiative processes associated with melanin-derived materials (30–39). Nanoparticles exhibiting high optical absorption capabilities—such as those doped with ICG/Mn^2+^—also facilitate efficient electron/charge transfer processes (40–42); their photothermal performance is comparable to that observed with CoF nanoparticles and polyoxyethylene ether nanoparticles (43–45). With reliable PA/MR signals, commendable light stability, and elevated PCE values, IMPP-c presents promising potential for further exploration *in* both *vitro* and *vivo* studies.

### 3.3 Cellular uptake, dark toxicity, and phototoxicity

**Figure 4:**
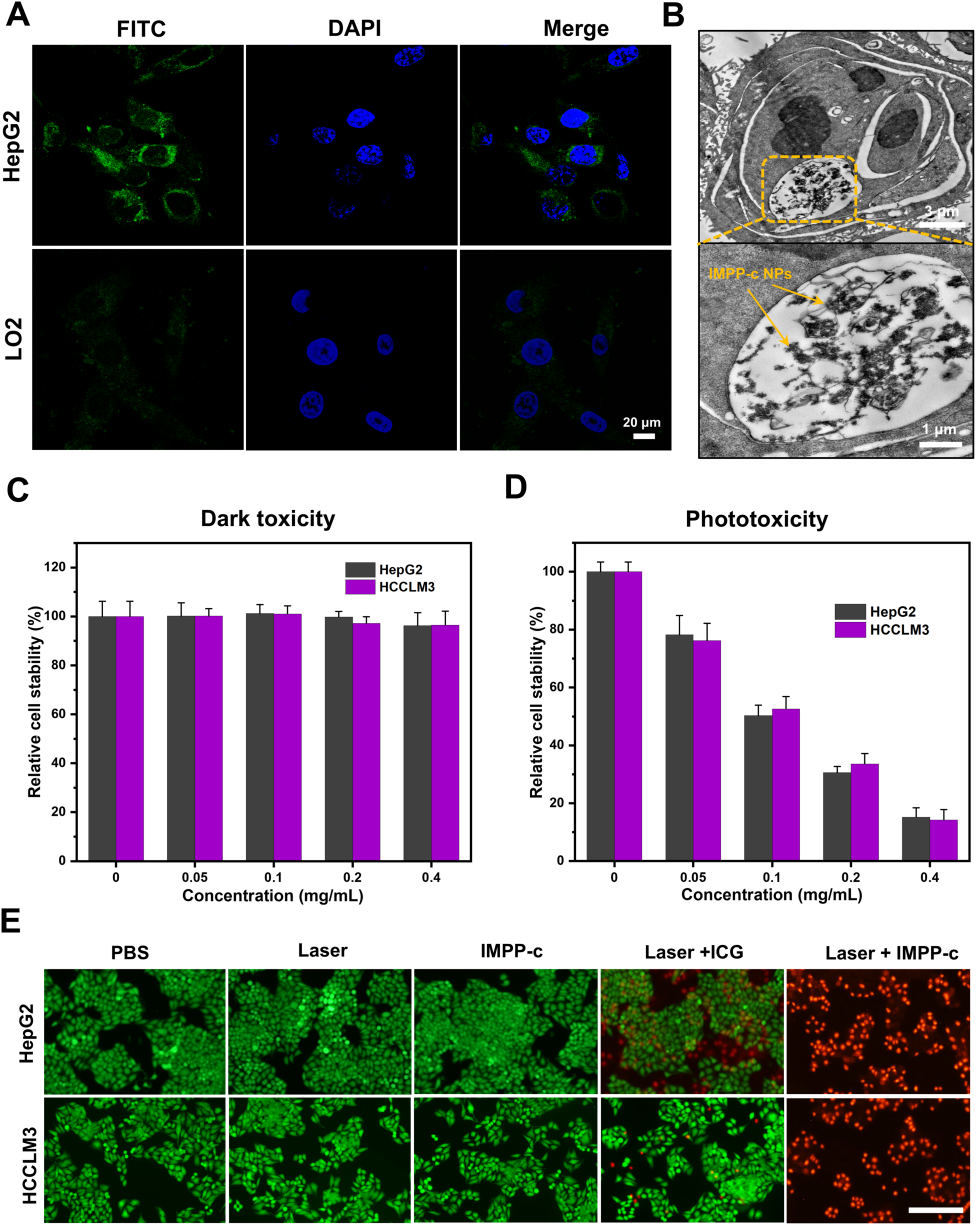
Cellular uptake and dark/photo toxicity in cell lines. **(A)** Confocal laser scanning microscopy images of HepG2 and LO2 cells treated with IMPP-c solutions for 4 hours. The cytoplasm exhibits green fluorescence from IMPP-c, while the cell nuclei are stained blue with DAPI, as shown in the merged image. **(B)** TEM images of HepG2 cells incubated with IMPP-c NPs. **(C)** Cell viability assessments of HepG2 and HCCLM3 cells following incubation with varying concentrations of IMPP-c NPs in darkness for 24 hours. **(D)** Cell viability evaluations of HepG2 and HCCLM3 cells after incubation with different concentrations of IPP-c NPs under laser irradiation for 10 minutes at a wavelength of 808 nm and an intensity of 0.8 W·cm^-2^. **(E)** Double staining images using Calcein AM (green, indicating live cells) and PI (red, indicating dead cells), depicting HepG2 and HCCLM3 cells treated with PBS, ICG, and IMPP-c both with and without laser irradiation.

In a recent series of research advancements, rigorous and comprehensive experiments have scientifically demonstrated that the CXCR4 protein is highly expressed on the cell membrane of HCC cells, including the HepG2 and HCCLM3 cell lines (46–48). Consequently, *in vitro* studies demonstrated that the overexpression of CXCR4 in HepG2 cells significantly enhanced their ability to actively internalize IMPP-c NPs. As illustrated in **Figures 4A** and **4B**, CLSM and TEM imaging clearly reveal substantial accumulation of CXCR4-modified (Alexa Fluro@488) IMPP-c NPs at both the cell membrane and within the cytoplasm of HepG2 cells after incubation with IMPP-c NPs for up to 4 hours. In contrast, LO2 cell lines incubated with IMPP-c NPs for an equivalent duration exhibited nearly undetectable levels of targeted uptake for this substance, suggesting the safety of normal human hepatocyte cells of the nanomaterials. This observation suggests that HCC cells possess specific uptake capabilities, underscoring the significant advantages offered by IMPP-c in this context. Therefore, it emerges as a promising candidate for targeted tumor diagnosis and treatment facilitated by *in vivo* biomedical imaging.

The cellular cytotoxicity of IMPP-c NPs was evaluated to assess their biocompatibility. As shown in **Figures 4C** and **Supplementary Figure S2** in the **Supporting Information**, after co-culturing IMPP-c NPs (0.4 mg·mL^-1^) with HepG2, HCCLM3 and LO2 cells in the absence of light for 24 hours, the survival rates for both cell types were found to exceed 91.2%, 92.5% and 96.8%, respectively, indicating that their cytotoxicity is negligible. Following this, a cellular phototoxicity test was conducted to confirm the efficacy of PTT and PDT using IMPP-c NPs under irradiation from an 808-nm laser for a duration of 10 minutes at an energy density of 0.8 W·cm^-^². **Supplementary Figure S3** in the **Supporting Information** illustrates the ROS generation characteristics of both IMPP-c NPs and ICG upon exposure to the 808-nm laser as detected by the DCFH-DA probe, further validating the PDT action of IMPP-c NPs. **Figure 4D** demonstrates that an increase in NP concentration corresponds with a decline in survival rates among HepG2 and HCCLM3 cells; specifically, following laser irradiation at a concentration of NPs-0.4 mg·mL^-1^, HepG2 cell viability decreased to approximately 13.2%, while HCCLM3 cell viability fell to around 11.6%. These results indicate significant phototoxic effects associated with increased NP concentrations. The photothermal effect on HepG2 and HCCLM3 cells was directly observed through Calcein-AM and PI dual staining experiments (**Figure 4E**). In both the HepG2 and HCCLM3 groups treated with PBS or subjected to either laser treatment or IMPP-c treatment alone, strong green fluorescence signals indicative of viable cells were observed. However, following the combined treatment with laser and ICG, a robust green fluorescence signal was noted alongside minimal red fluorescence signals representing dead cells. In contrast, significant red fluorescence signals were observed following treatment with laser and IMPP-c NPs. These results indicate that the targeted delivery utilizing IMPP-c NPs can effectively engage HCC cells through PTT and PDT modalities, while enabling appropriate adjustments in laser intensity based on its targeting capabilities combined with low toxicity profiles *in vitro*. Consequently, due to its selective targeting ability alongside its advantageous PTT/PDT characteristics, IMPP-c is considered a reliable and specific therapeutic agent for the treatment of HCC.

### 3.4 *In vivo* PA/MR imaging of subcutaneous tumors

**Figure 5:**
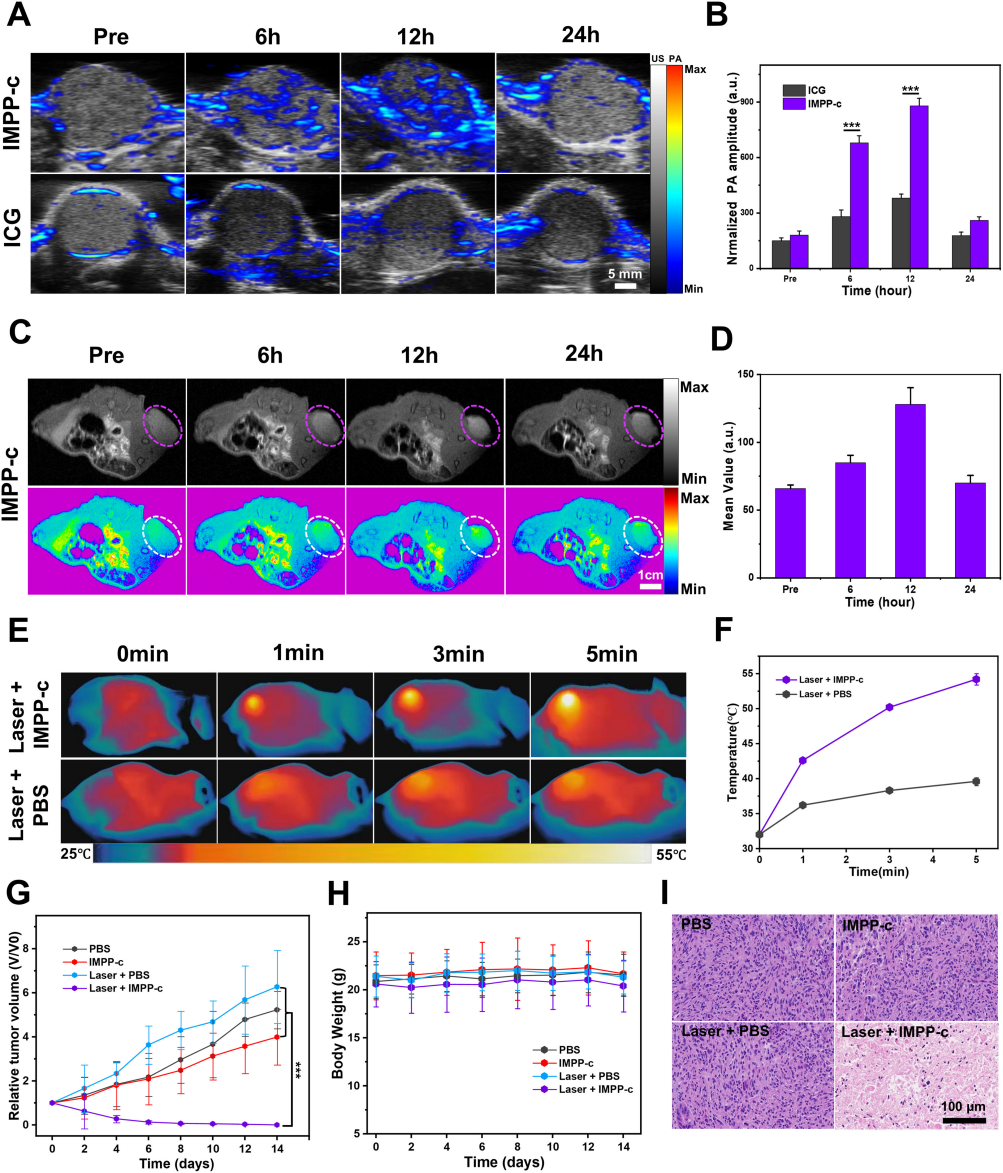
*In vivo* PA/MR imaging and phototherapy of subcutaneous tumors. **(A)** *In vivo* PA imaging of subcutaneous tumors at various post-injection time points following the administration of IMPP-c NPs and ICG (Excitation wavelength: 808 nm). **(B)** Corresponding quantification of PA signals at the tumor site in both the IMPP-c NPs and ICG injection groups. **(C)** *In vivo* MR images of mice bearing subcutaneous tumors after receiving injections of IMPP-c NPs. **(D)** Quantitative analysis of average MR signal intensities. **(E)** Infrared thermal imaging of subcutaneous HepG2-tumor-bearing mice treated with either PBS or IMPP-c, subjected to 808-nm laser irradiation (0.8 W·cm^−2^) for varying durations. **(F)** Heating curves recorded at the tumor site during laser irradiation. **(G)** Tumor growth curves (n = 4, mean ± SD) across different treatment groups. **(H)** Changes in body weight among nude mice within various treatment cohorts (n = 4). **(I)** Histological microscopy images depicting tumors after treatments administered across different groups over a period of 15 days. ^***^ *P*<0.001.

Because IMPP-c has demonstrated effective targeting capabilities *in vitro*, a PA and MR imaging study was conducted on a subcutaneous HepG2 tumor model to validate the tumor-specific accumulation characteristics of IMPP-c *in vivo*. A dose of 30 mg·kg^-1^ of IMPP-c NPs combined with ICG, at equal dosage, was administered *via* the tail vein. The PA signal within the tumor region was monitored in real-time using a PA imaging system over the course of 24 hours post-administration. As illustrated in **Figure 5A**, NPs effectively targeted and accumulated within the tumor region at 12 hours following injection in the IMPP-c group; conversely, no significant aggregation was observed in the ICG group. Quantitative analysis of the PA signal from the tumor region, as depicted in **Figure 5B**, revealed that both at 6 and 12 hours after injection, the PA signal intensity for the IMPP-c group was significantly greater than that for the ICG group (*P*<0.001). Furthermore, at 24 hours post-injection, there was a notable enhancement of PA signal intensity within the tumor region for the IMPP-c group compared to that of ICG. IMPP-c possesses an appropriate size along with established biocompatibility and targeted homing capabilities toward tumors, enabling effective accumulation at tumor sites. Additionally, these accumulation characteristics were corroborated through MR imaging techniques. The subcutaneous mouse tumors were imaged utilizing Turbo RARE T_1_ imaging sequences (field of view: 3 cm; slice thickness: 0.5 mm). As depicted in **Figures 5C** and **5D**, average values within the tumor region gradually increased up to 12 hours following injection before diminishing by 24 hours—results consistent with those obtained from PA imaging. In conclusion, we recommend employing IMPP-c for efficient PA imaging as well as MR imaging-guided precise theranostic applications *in vivo*.

### 3.5 *In vivo* PTT/PDT of subcutaneous tumors

Under the precise guidance of PA and MR imaging, targeted PTT/PDT for a subcutaneous HepG2 tumor model were scheduled to be conducted 12 hours post-injection of IMPP-c NPs. As illustrated in **Supplementary Figure S4** in the supporting information, details regarding the NIR PTT/PDT device and its configuration are provided. The PTT/PDT treatment utilizing IMPP-c NPs is depicted in **Figures 5E** and **5F**. Under real-time accurate guidance from both an 808-nm laser (energy density: 0.8 W·cm^-^²) and optical imaging within the Laser + IMPP-c group, tumor region temperatures surged to approximately 53.8°C within just 5 minutes while being maintained at elevated levels for 8 minutes thereafter. In contrast, temperatures within the tumor region treated with Laser + PBS only reached about 39.5°C. Consequently, owing to the PTT/PDT effects induced by IMPP-c NPs—exceeding thresholds above 53.8°C—tumor tissues can be effectively ablated without significantly affecting surrounding healthy tissues containing minimal amounts of IMPP-c NPs that remained below approximately 39.5°C; thus minimizing collateral damage to normal tissue. The tumor growth curves and corresponding digital images of the mice are presented in **Figures 5G** and **Supplementary Figure S5** in the **Supporting Information**. As anticipated, throughout the entire study period of 15 days, tumors in the laser + IMPP-c group were completely eradicated, while no significant inhibition of tumor growth was observed in the other groups (laser + PBS, PBS, and IMPP-c). The body weight across all groups remained stable during the treatment period (**Figure 5H**). Furthermore, pronounced necrosis of tumor tissue was evident in H&E-stained sections from mice treated with laser + IMPP-c; conversely, there were no notable signs of apoptosis or tissue necrosis in tumor cells from mice treated with laser + PBS, PBS alone, or IMPP-c (**Figure 5I**). The successful therapeutic outcomes observed in subcutaneous tumor models may serve as a catalyst for further investigations into treatments and diagnostics related to orthotopic SHCC models facilitated by assistance from IMPP-c.

### 3.6 *In vivo/ex vivo* PA/MR imaging of orthotopic SHCCs

**Figure 6:**
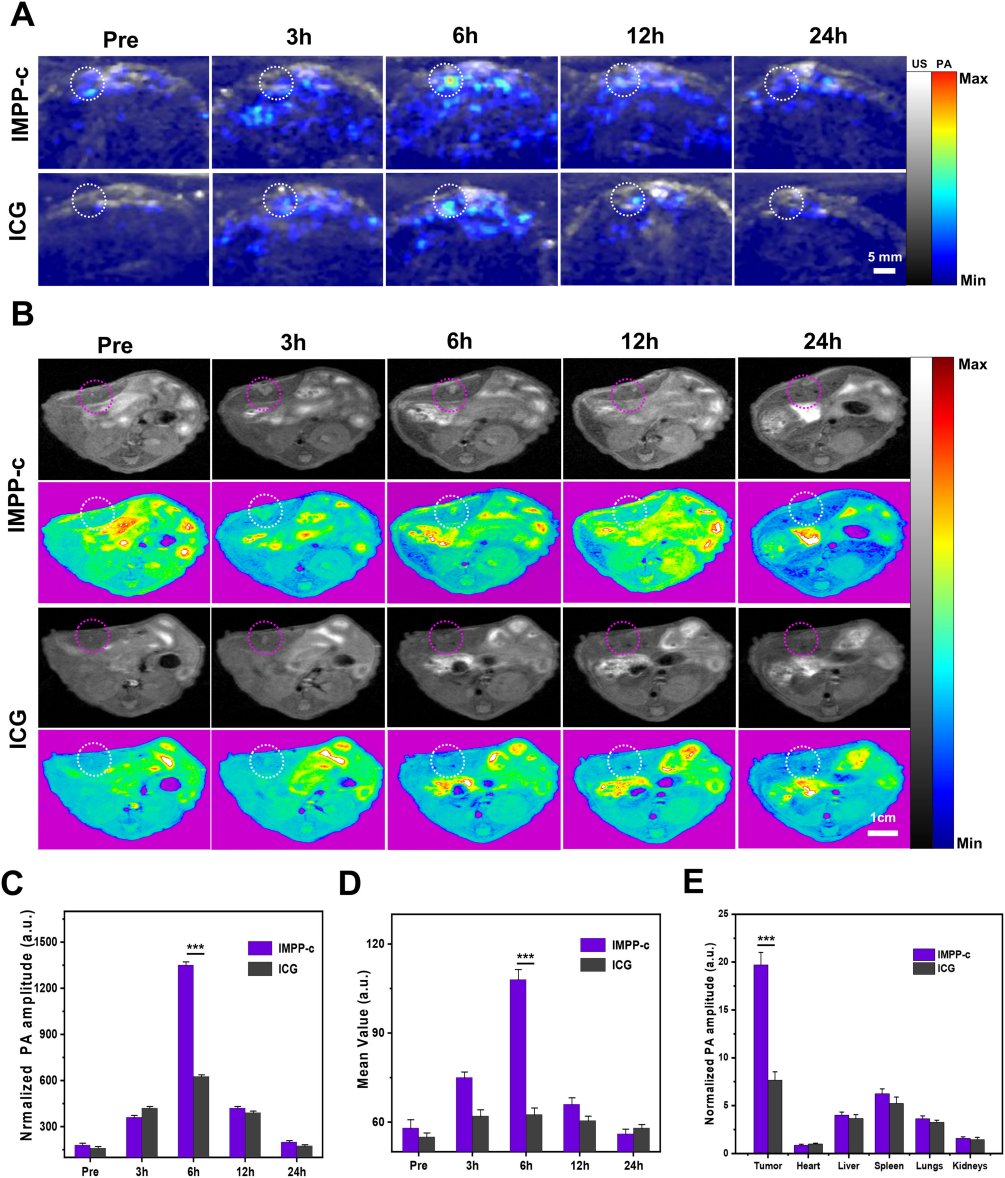
*In vivo* PA/MR imaging of orthotopic tumors. **(A)** *In vivo* PA imaging of the orthotopic tumor at various post-injection time points following administration of IMPP-c NPs and ICG (Excitation wavelength: 808 nm). **(B)** *In vivo* MR imaging of the orthotopic tumor at different post-injection time intervals for IMPP-c NPs and ICG. **(C)** Corresponding quantification of PA signals at the tumor site from **(A)**. **(D)** Quantitative analysis of average MR signal intensities from **(B)**. **(E)** Quantification of mean *ex vivo* PA signals from the liver tumor and major organs 6 hours after injection of IMPP-c NPs/ICG. ^***^ *P*<0.001.

In prior *in vitro* and *in vivo* studies, the targeting efficacy of IMPP-c NPs has been rigorously validated. To evaluate the performance of PA imaging utilizing these NPs in abdominal tumors, an orthotopic SHCC mouse model was employed for PA imaging assessment. Following a 10-day period post-cell implantation, the establishment of SHCC in nude mice was confirmed *via* biological fluorescence monitoring. Subsequently, IMPP-c NPs conjugated with ICG at a dosage of 30 mg·Kg^-1^ were administered through intravenous injection into the nude mice; optimal tumor imaging regions were then assessed using a NIR PA imaging system within 24 hours post-injection. As depicted in **Figure 6A**, within the IMPP-c group, NPs exhibited effective targeting and significant accumulation within the tumor region as early as 6 hours following injection; conversely, no substantial accumulation was observed in the ICG group. Furthermore, quantitative analysis depicted in **Figure 6C** indicated that PA signal intensity within the tumor region for the IMPP-c group was considerably greater than that observed for the ICG group (*P*<0.001) at the 6-hour mark post-injection. *Ex vivo* PA imaging conducted at this time point further verified NPs accumulation across various major organs and tumors, as shown in **Figure 6E**. The PA signal corresponding to orthotopic tumors exhibited substantially higher intensity within the IMPP-c group compared to that within the ICG group (*P*<0.001), confirming targeted aggregation of IMPP-c NPs specifically at orthotopic SHCCs.

In previous studies, orthotopic SHCC has been imaged using a PA imaging system to monitor cross-sections of the tumor. However, *in vivo* MR imaging can also visualize the distribution throughout the body following the injection of IMPP-c NPs/ICG. Specifically, IMPP-c NPs conjugated with ICG were administered to nude mice *via* intravenous injection at a dosage of 30 mg·kg^−1^. The optimal region for tumor imaging was assessed using an MR imaging system within 24 hours post-injection. As demonstrated in **Figure 6B**, within the IMPP-c group, NPs exhibited targeted accumulation in the tumor region as early as 6 hours after administration; in contrast, no significant accumulation was observed in the ICG group. Furthermore, as demonstrated in **Figure 6D**, average signal values within the tumor area gradually increased over a period extending up to 6 hours post-injection and were significantly greater than those observed in the ICG group (*P*<0.001), which aligns with findings from PA imaging. These results—both *in vivo* and *ex vivo*—indicate that the targeted nanocarrier IMPP-c is actively taken up by CXCR4-overexpressing SHCC tumor cells and accumulates within tumoral tissues; whereas ICG is rapidly metabolized and fails to be absorbed by tumor cells. Therefore, we recommend the utilization of IMPP-c for effective PA/MR imaging, in conjunction with imaging-guided precision theranostics for SHCC *in vivo*.

### 3.7 *In vivo* noninvasive PTT/PDT of orthotopic SHCCs

**Figure 7:**
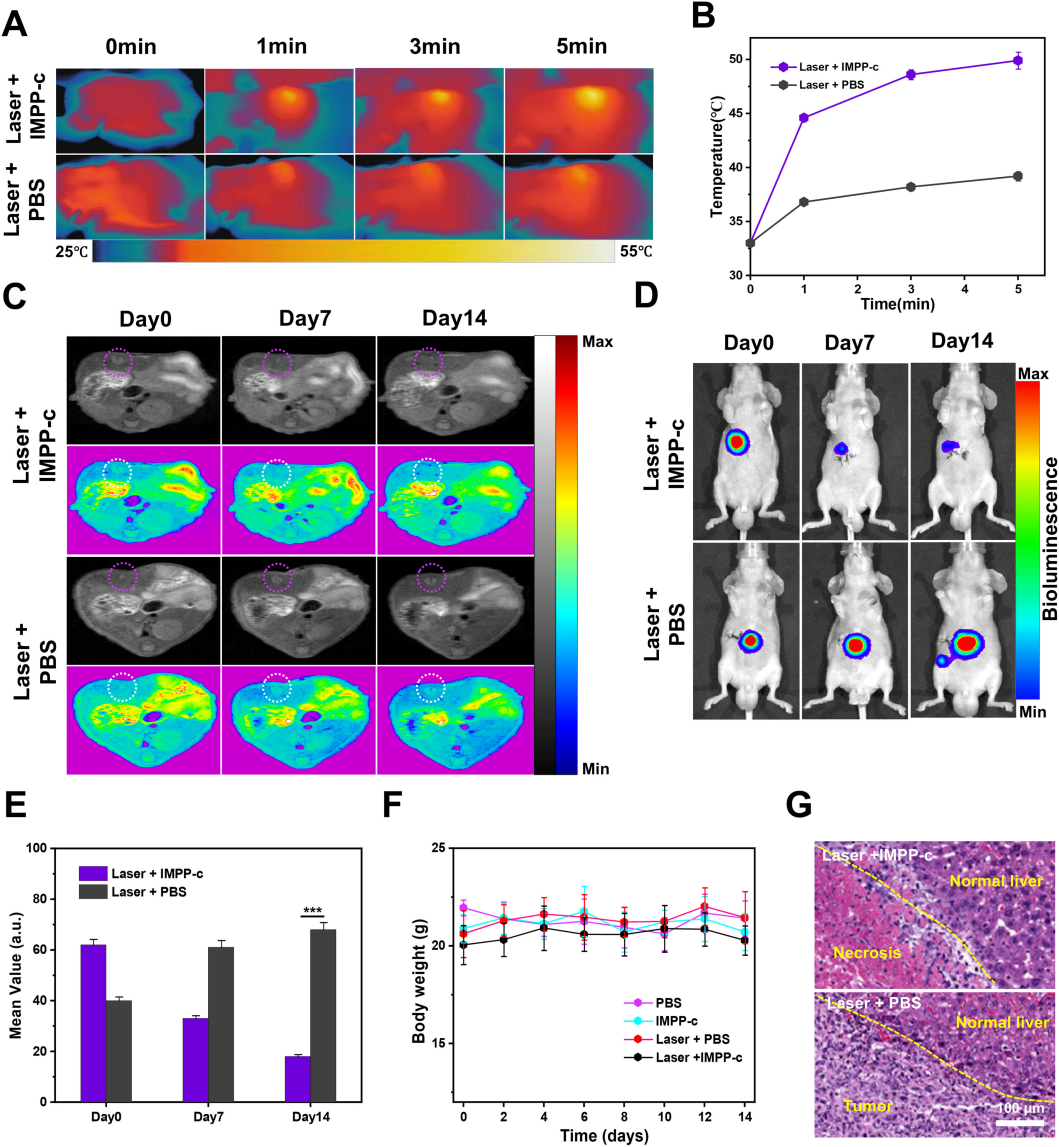
*In vivo* PA/MR imaging-guided phototherapy of orthotopic tumors. **(A)** Infrared thermal imaging was conducted on orthotopic SHCC tumor-bearing mice treated with either IMPP-c or PBS, followed by 808-nm laser irradiation (0.8 W·cm⁻²) for varying durations. **(B)** Heating curves of the tumor sites in both groups during laser irradiation were recorded. **(C)** Continuous monitoring of the MR signals from orthotopic SHCCs in both groups was performed before and after phototherapy at Pre, Day 7, and Day 14. **(D)** Bioluminescence signals from orthotopic SHCCs were continuously monitored in both groups before and after phototherapy at Pre, Day 7, and Day 14. **(E)** Relative MR signal changes of orthotopic SHCCs derived from **(C)**. **(F)** Changes in body weight among mice across different treatment groups are illustrated (n = 4). **(G)** H&E staining results of SHCCs dissected from treated mice in both groups are presented, collected 15 days post PTT/PDT treatment. ^***^ *P*<0.001.

Under the guidance of PA/MR imaging, the optimal time point for treating orthotopic SHCC using PTT and PDT has been determined to be 6 hours post-injection. Previous studies have indicated that the depth of SHCC within the abdominal cavity is approximately 4 mm (49), which can be effectively penetrated by an 808-nm laser with minimal attenuation (50–52) (refer to **Supplementary Figure S6** in the **Supporting Information**). Prior to treatment, a PTT experiment was conducted on IMPP-c, whole blood, PBS, and normal liver tissue obtained *via* laparotomy to evaluate the safety profile of PTT (see **Supplementary Figure S7** in the **Supporting Information**). As anticipated, following irradiation with an 808-nm laser at an energy density of 0.8 W·cm⁻², the temperature of IMPP-c exhibited a rapid increase of 26.3°C within just 10 minutes. In contrast, temperatures for whole blood, liver tissue, and PBS only increased by 3.4°C, 3.0°C, and 2.8°C respectively—demonstrating reliable biocompatibility. **Figures 7A** and **7B** depict PTT/PDT treatments for SHCC utilizing IMPP-c NPs; here it is evident that real-time accurate optical imaging guided under irradiation from an 808-nm laser resulted in a swift elevation of tumor region temperatures to around 50°C while maintaining this elevated temperature for a duration of 8 minutes. Such high temperatures are effective for tumor ablation without inflicting secondary damage upon surrounding healthy tissues. Conversely, temperatures in the tumor region within both laser + PBS groups reached only approximately up to 40°C. As demonstrated through both *in vivo* and *in vitro* PA/MR imaging investigations highlighting their active and passive targeting capabilities, IMPP-c NPs achieve significantly high concentrations within tumorous lesions while exhibiting low concentrations in normal liver tissues. Consequently, the noninvasive PTT/PDT results in effective elimination of tumor cells due to heat application (exceeding temperatures above 50°C) localized at areas rich with IMPP-c NPs; meanwhile normal liver tissue containing sparse amounts yields negligible phototherapy effects (remaining below or around capacities like under ~40°C), thereby minimizing phototherapeutic damage inflicted upon healthy tissues.

**Figures 7C** and **7D** illustrate the findings from evaluating the effectiveness of noninvasive PTT/PDT in orthotopic tumor models, utilizing MR and biofluorescence imaging. The MR/biofluorescence imaging results demonstrate a significant reduction in signal within the tumor area of the laser + IMPP-c group, whereas no notable change is observed in the tumor area of the laser + PBS group. As depicted in **Figure 7E**, there is a gradual decrease in the average signal within the tumor area for the laser + IMPP-c group, while no significant alteration occurs in the laser + PBS group. Additionally, as illustrated in **Supplementary Figure S8** (**Supporting Information**), quantitative data obtained from biofluorescence imaging indicate a significant reduction in biofluorescence signals within the tumor region for the laser + IMPP-c group. In contrast, an increase in biofluorescence signals is observed in tumors treated with laser + PBS. Given that cell survival rates directly correlate with orthotopic SHCC outcomes, these promising MR/biofluorescence imaging results support light-based therapies as effective treatments for SHCC. Throughout the treatment period, weight measurements across all groups remained stable (refer to **Figure 7F**). **Figure 7G** presents H&E staining results and measurements of tumor diameter across all groups 15 days post-treatment. The laser + IMPP-c cohort exhibited marked tumor necrosis and suppression following phototherapy, while notable proliferation without necrosis was evident in tumors from the laser + PBS group after treatment. These findings further validate the efficacy of utilizing IMPP-c NPs to specifically target SHCCs for noninvasive PTT/PDT. According to existing literature reports, comparable studies conducted on mice have demonstrated successful outcomes with phototherapy targeted at SHCCs; however, non-targeted or imprecise treatments are less than optimal. Therefore, targeted approaches utilizing noninvasive methods combined with accurate optical imaging guidance provide an innovative strategy for PTT/PDT interventions applied to orthotopic SHCCs in mice—a method that holds considerable promise for clinical translation potential.

### 3.8 Metabolic clearance of IMPP-c NPs *in vivo*

To further explore the metabolic clearance of IMPP-c NPs *in vivo*, we examined the distribution of these particles within major organs—including the heart, liver, spleen, lungs, and kidneys—at 24, 36, and 48 hours following intravenous injection. As a control measure, an equivalent volume of PBS was administered to a separate cohort of mice to establish baseline PA signals in each organ. **Supplementary Figure S9** in the **Supporting Information** demonstrates that relative PA signals in major organs diminished progressively during the first 24 hours post-injection and eventually disappeared within 48 hours when compared to the control group. Considering their effective tumor-targeting capabilities and favorable metabolic clearance properties, we conclude that IMPP-c NPs presents itself as a promising contrast agent for *in vivo* tumor diagnosis and therapy employing PA imaging techniques.

### 3.9 Biosafety of IMPP-c NPs

For successful clinical translation, it is crucial to conduct a comprehensive evaluation of the safety profile associated with phototherapy. The potential for tissue damage or harm to vital organs arising from PTT and PDT can be systematically assessed utilizing various methodologies: monitoring NPs metabolism, recording body weight and vital signs, evaluating biochemical indicators, and performing histological examinations of organs post-surgery. Importantly, the PA signal in liver, spleen, heart, lung, and kidney tissues returned to baseline levels within 48 hours following NPs injection. This observation indicates that IMPP-c NPs are effectively metabolized *in vivo*. We continuously monitored the weight and vital signs across all groups for 14 days after treatment. No significant abnormal behaviors or weight loss—such as unusual movements, aggression, or restlessness—were observed in any group. Furthermore, H&E staining results for major organs at day 15 post-treatment are presented in **Supplementary Figure S10** (**Supporting Information**). There was no evident tissue or organ damage detected in any group during this period; thus confirming the safety throughout the entire treatment course involving NIR laser irradiation and NPs injection. Additionally, we evaluated the biocompatibility of IMPP-c NPs and phototherapy by measuring routine blood parameters and biochemical indicators in nude mice across all groups. No significant abnormalities were identified in these assessments (**Supplementary Figure S11** in **Supporting Information**), suggesting that IMPP-c NPs exhibit excellent biocompatibility *in vivo*. The targeted nanocarrier IMPP-c has demonstrated commendable stability and biocompatibility along with superior PA/MR imaging capabilities and reliable noninvasive PTT/PDT performance. This positions it favorably for advancing nanomedicine applications related to photothermal therapy across various tumor models. In conclusion, our findings suggest that this nanoplatform, when integrated with imaging-guided phototherapy, has significant potential for precise diagnosis and effective treatment of HCC.

## 4 Conclusions

In summary, a highly biocompatible and multifunctional nanoplatform, designated as ICG/Mn-PDA-PEG-CXCR4 (IMPP-c), has been meticulously developed for the diagnosis and treatment of orthotopic SHCC through PA/MR imaging-guided noninvasive PTT/PDT. *In vitro* experiments demonstrated that IMPP-c NPs exhibited targeted accumulation properties in HepG2 cells. Significantly, the dual-modal PA/MR imaging capabilities of IMPP-c facilitated high-resolution visualization alongside deep tissue penetration, thereby enabling precise localization of early-stage orthotopic SHCC lesions. *In vivo* experiments utilizing PA/MR imaging-guided noninvasive PTT/PDT revealed that SHCC was non-invasively eradicated completely without any signs of recurrence. Additionally, metabolic processes involving IMPP-c were observed across major organs during treatment, underscoring its favorable biocompatibility profile. This study introduces an innovative strategy for diagnosing and implementing non-invasive therapeutic interventions using nanoparticle systems such as IMPP-c, thus establishing a foundation for further clinical applications.

## Data Availability

The original contributions presented in the study are included in the article/**Supplementary Material**. Further inquiries can be directed to the corresponding authors.
