# Supplementary material for: PA/MR imaging-guided precision phototherapy and efficacy evaluation of hepatocellular carcinoma utilizing a targeted multifunctional nanoprobe
Source: Front Immunol. 2025 Jun 11;16:1605048. doi: 10.3389/fimmu.2025.1605048 (PMC12187729; doi:10.3389/fimmu.2025.1605048)
Supplement: Supplementary file 1 [file DataSheet1.pdf]

Supporting Information

PA/MR Imaging-Guided Precision Phototherapy and Efficacy Evaluation of Hepatocellular Carcinoma Utilizing a Targeted Multifunctional Nanoprobe

Shuo Qi, Lei Zhou, Weinan Liu, Lian Shen, Yuping Yao, Bingzhang Tian, Changjun Liu*, Wei Cheng*, Kang Chen*

***Corresponding author:**

Kang Chen, MD., Department of Hepatobiliary Surgery, Hunan Provincial People's Hospital, The First Affiliated Hospital of Hunan Normal University, Changsha, 410005 Hunan Province, China. Email: chenkang@hunnu.edu.cn.

Wei Cheng, PhD., Department of Hepatobiliary Surgery, Hunan Provincial People's Hospital, The First Affiliated Hospital of Hunan Normal University, Changsha, 410005 Hunan Province, China. Email: chengwei@hunnu.edu.cn.

Changjun Liu, PhD., Department of Hepatobiliary Surgery, Hunan Provincial People's Hospital, The First Affiliated Hospital of Hunan Normal University, Changsha, 410005 Hunan Province, China. Email: liuchangjun@hunnu.edu.cn.

1. Synthesis of IMPP-c NPs

First, an aqueous solution of dopamine hydrochloride (5 mg·mL^{-1}, 3 mL) was added to a 0.2 M $NaHCO_3$-HCl buffer (25 mL, pH 8.2). Subsequently, 4 mL of $MnCl_2$ (25 mM) was introduced while maintaining stirring at room temperature for 2 hours. Following this, 0.5 mg of ICG stock solution (5 mg·mL^{-1}) was incorporated into the aforementioned mixture and stirred at room temperature for an additional 6 hours. The resulting solid product (ICG/Mn-PDA) was then collected by centrifugation at 10,000 × g and washed five times with deionized water. Second, the nano powders were dispersed in 2 mL of deionized water via sonication and subsequently added to a PEG-SH solution (10 mL; concentration: 2 mg·mL^{-1}), allowing for PEG capping over a period of 24 hours. Finally, CXCR4 antibody (20 μg·mL^{-1}) was introduced into the particle suspension and allowed to react at room temperature in the dark for 8 hours. Afterward, the mixture underwent centrifugation and washing with deionized water three times before being lyophilized to yield a black solid powder comprising ICG/Mn-PDA-PEG-CXCR4 nanoparticles (IMPP-c NPs).

2. The evaluation of photothermal conversion efficiency (PCE) for IMPP-c

The PCE for IMPP-c (η) was calculated following the method as reported [1]:

$$\eta = \frac{hS(T_{Max}-T_{Surr})-Q_{Dis}}{I(1-10^{-A_{808}})} \quad (1)$$

η refers to the conversion efficiency form 808-nm laser energy to thermal energy. T_{Max} is the equilibrium temperature and the T_{Surr} is the ambient temperature of the surroundings. Q_{Dis} expresses the baseline energy generated by sample cell and water upon laser irradiation. *I* is the incident laser power. A_{808} is the absorbance of IMPP-c at 808 nm. *h* and *S* respectively represent the heat transfer coefficient and surface area of the cell. *m* and *c* refer to the mass and capacity of pure water. hS is calculated from substituting equations:

$$\theta = \frac{T-T_{Surr}}{T_{Max}-T_{Surr}} \quad (2)$$

θ is the driving force temperature of IMPP-c.

$$t = -\tau_s ln\theta \quad (3)$$

τ_S is the time constant.

$$hS = \frac{\mathrm{mc}}{\tau_S} \quad (4)$$

The m was weighted to be 1 mg and c is 4.2 mJ·mg^{-1}°C^{-1}. τ_S is 198 s. T_{Max}-T_{Surr} is 25.2 °C. Q_{Dis} was measured to be 79.4 mW. The absorbance A_{808} was 0.7885, and I is 800 mW. In conclusion, the PCE of IMPP-c is calculated to be 67.9%.

3. Animal models

The experimental animals utilized in this study were BALB/c nude mice, which were provided by Hunan SJA Experimental Animal Co., Ltd. All animal experiments received approval from the Experimental Animal Ethics Committee of Hunan Provincial People's Hospital. Approximately 1×10^6 HepG2 cells were injected into the subcutaneous tissue of the left thigh of BALB/c nude mice using a mixture of 100 μL (PBS: matrix gel=1:1) to establish a subcutaneous hepatocellular carcinoma (HCC) model for subsequent experiments. The tumor volume targeted for further experimentation was approximately 80 mm³. Additionally, around 6×10^5 HepG2 cells were injected into the liver lobe of BALB/c nude mice utilizing a mixture of 50 μL (PBS: matrix gel=1:1) to create an orthotopic HCC model. In this orthotopic HCC model, each tumor was implanted at the same anatomical location to facilitate management during subsequent experiments. Prior to injection, HepG2 cells were pre-expressed with luciferase. 10 days post-injection, in *vivo* fluorescence imaging was conducted using D-luciferin potassium salt (200 μL, 15 mg·mL^{-1}), which was administered into the peritoneal cavity of the mice. The Xtreme Live Imaging System from Bruker (Germany) was employed to detect fluorescence emitted by the orthotopic tumors. Appropriate mice were selected for further experimentation based on these imaging results.

4. *In vivo* metabolism of IMPP-c NPs

The PA imaging system was employed to conduct *ex vivo* photoacoustic (PA) imaging of major organs, including the liver, spleen, heart, lungs, and kidneys, in rats. This was done to assess the clearance capability of IMPP-c NPs within the circulation of mice. Furthermore, to evaluate the biocompatibility of IMPP-c NPs and

phototherapy across different groups of mice, blood samples were collected from the tails of mice 15 days post-treatment via intravenous access for routine hematological and biochemical analyses. The averages were subsequently calculated.

5. Histopathology test

After the PTT/PDT treatment, tumors from all groups were subjected to hematoxylin and eosin (H&E) staining for histopathological examination. Additionally, major organs—including the heart, liver, spleen, lungs, and kidneys—were also stained with H&E to further assess the biosafety of IMPP-c.

6. Statistical Analysis

The data are presented as mean ± standard deviation (M ± SD), and statistical comparisons were conducted using the Analysis of ANOVA with post-hoc corrections as necessary. Statistical analysis and figure plotting were performed utilizing MATLAB software (MathWorks, USA) and Origin software (OriginLab, USA). *P* value of less than 0.05 was considered statistically significant.

References

[1] Wu W, Pu Y, Gao S, et al. Bacterial Metabolism-Initiated Nanocatalytic Tumor Immunotherapy[J]. **Nanomicro Lett.** 2022, 14(1): 220.

Table 1 Comparison of PCEs with other reported nanoparticles in the Near-Infrared Window.

**Name**	**Excitation wavelength**	**PCE**	**Reference**
IMPP-c	808 nm	67.9%	**Our work**
tfm-BDP NPs	808 nm	64.7%	**Adv Mater 2020,** 32(11):e1907855.
S-CDs	808 nm	55.4%	**Nanoscale 2021,** 13(34):14426-14434.
Bi_2S_3:Gd@Cu-BIF	808 nm	52.6%	**Acta Biomater 2022,** 143:445-458.
TPP-Bi@PDA@CP	808 nm	52.3%	**ACS Appl Mater Interfaces 2022,** 14(22):25050-25064.
Cu@CPP-*t*	808 nm	48.5%	**Small 2020**, 16(1): e1905184.
NaYF(4):Yb/Er/Nd @NaYF(4):Nd	808 nm	45%	**Bioact Mater 2022,** 17:71-80.
4-Pf-NPs	808 nm	41.8%	**J Am Chem Soc 2022,** 144(41):18908-18917.
CPPDA-Hf@Poloxamer	808 nm	33.3%	**Chem Commun (Camb) 2021**, 57(87): 11473-11476.
Os@Mucin	808 nm	22.83%	**Nano Lett 2024,** 24(45):14337-14345.

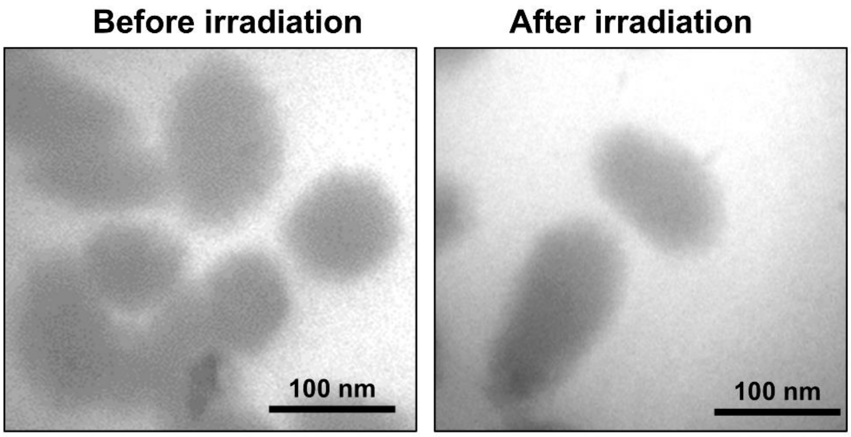

Figure S1. TEM images of IMPP-c before and after exposure to 808-nm laser irradiation.

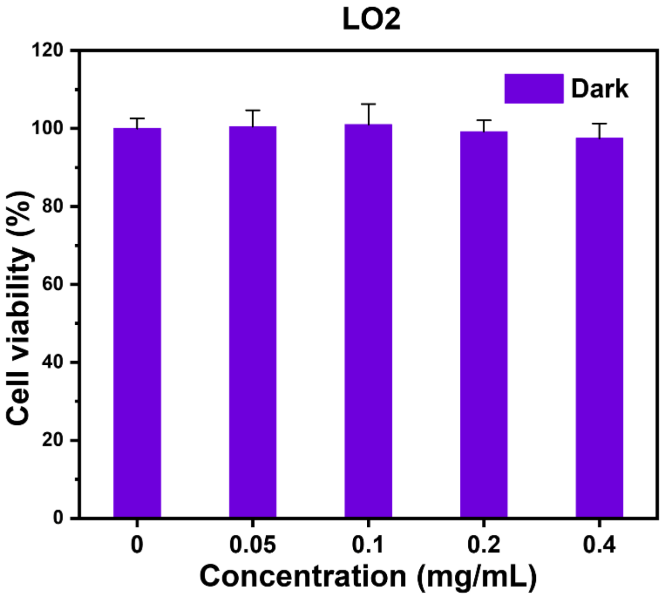

Figure S2. Cell viability assessments of LO2 cells following incubation with varying concentrations of IMPP-c NPs in darkness for 24 hours.

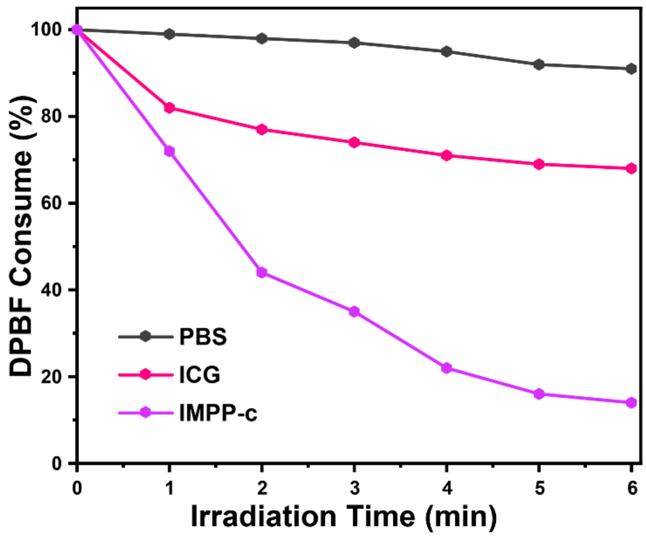

Figure S3. The DPBF consumption curve in the PBS, ICG, and IMPP-c groups under 808-nm laser irradiation.

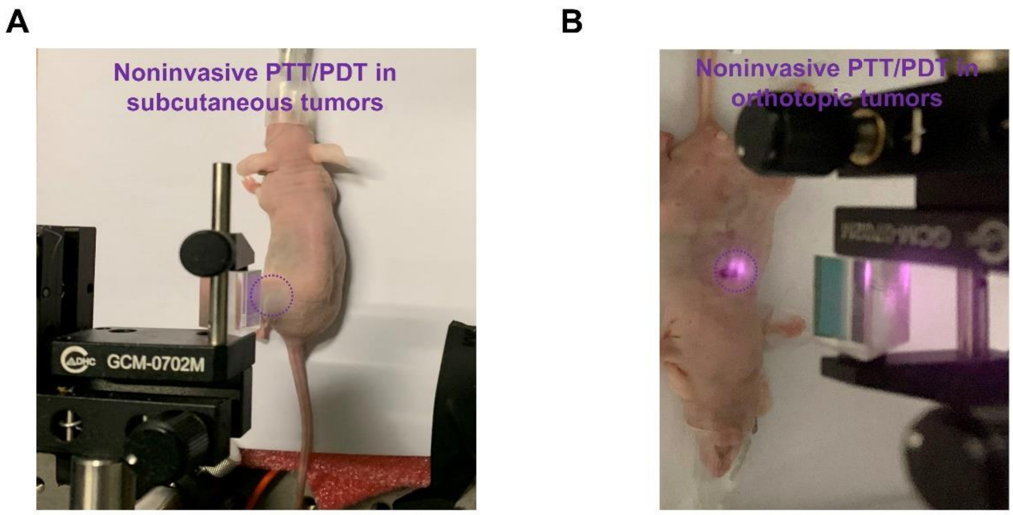

Figure S4. Diagram of PTT/PDT treatment device for subcutaneous tumors and orthotopic tumors.

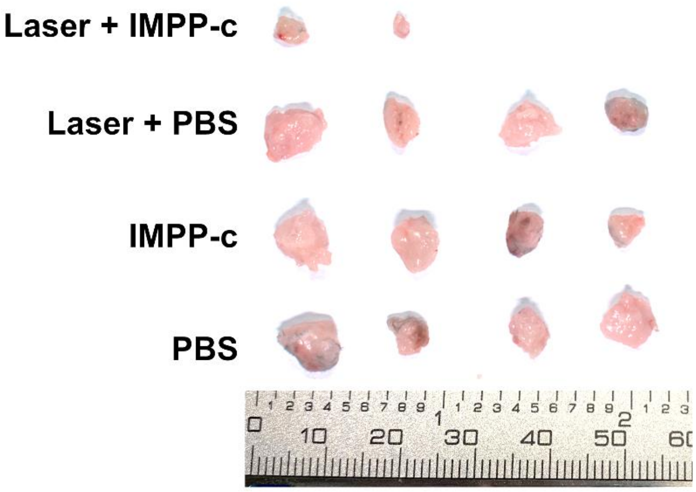

Figure S5. Photographs depicting the subcutaneous tumors in the Laser + IMPP-c, Laser + PBS, IMPP-c, and PBS groups (n = 4 per group).

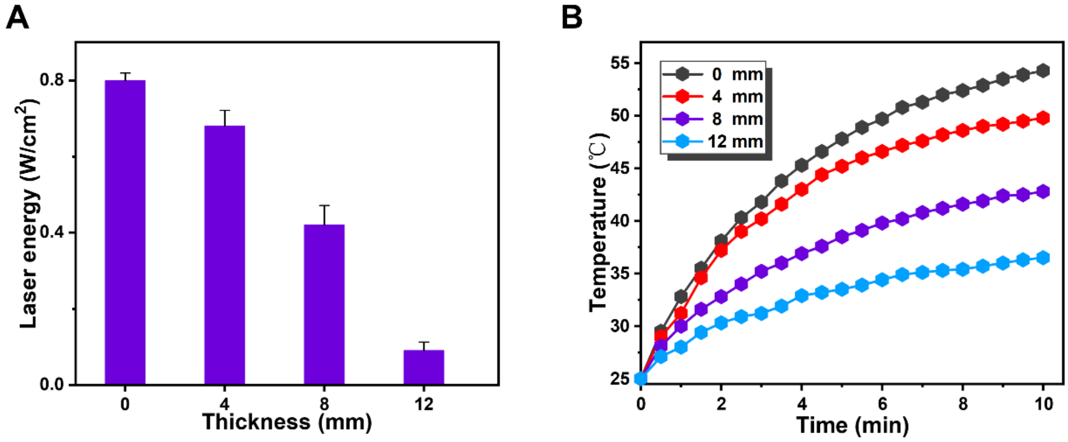

Figure S6. (A) The attenuation of laser penetration energy as it traverses various thicknesses of chicken tissues. (B) The photothermal heating effect of IMPP-c at different tissue depths, irradiated with an 808-nm laser at a power density of 0.8 W·cm^{-2}.

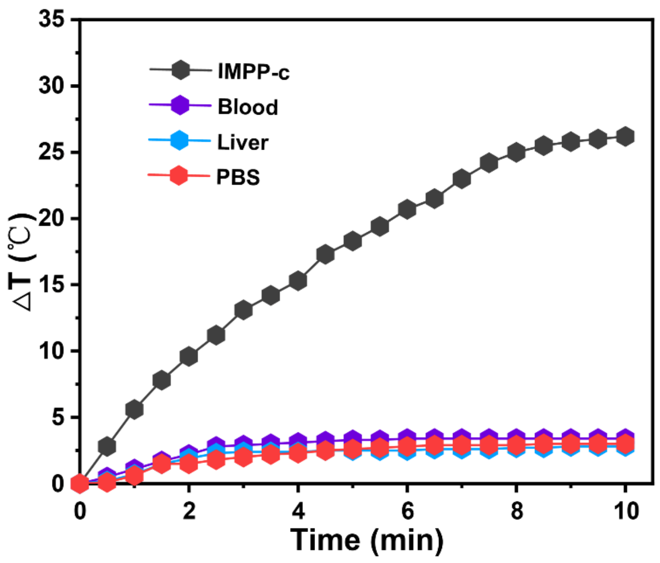

Figure S7. Temperature elevation curves for IMPP-c, blood, liver, and PBS during laser irradiation (808 nm, 10 minutes, 0.8 W·cm^{-2}).

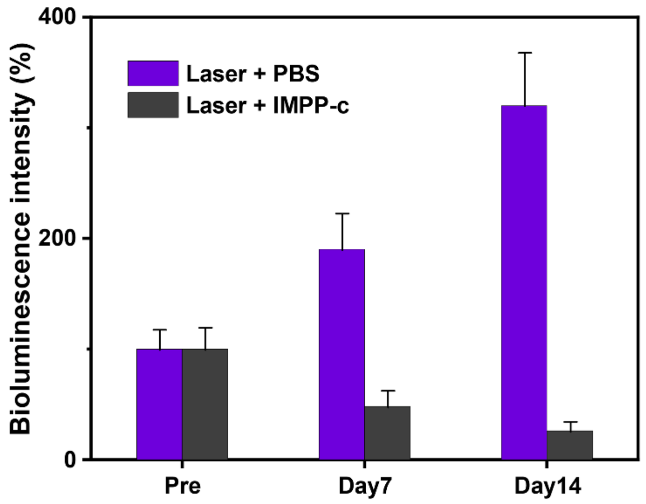

Figure S8. Relative bioluminescence monitoring of orthotopic HCCs was conducted before and after treatment in both groups, specifically at baseline, day 7, and day 14 (n = 4).

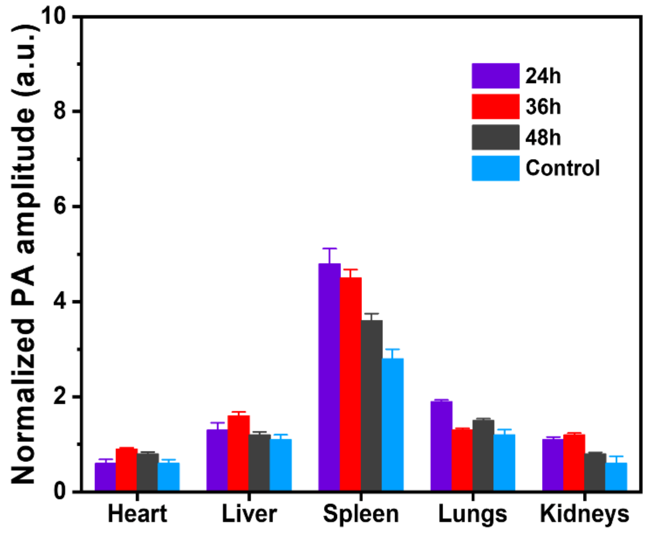

Figure S9. The PA signals from the major organs of tumor-free mice were recorded at 24, 36, and 48 hours following injection (n=3).

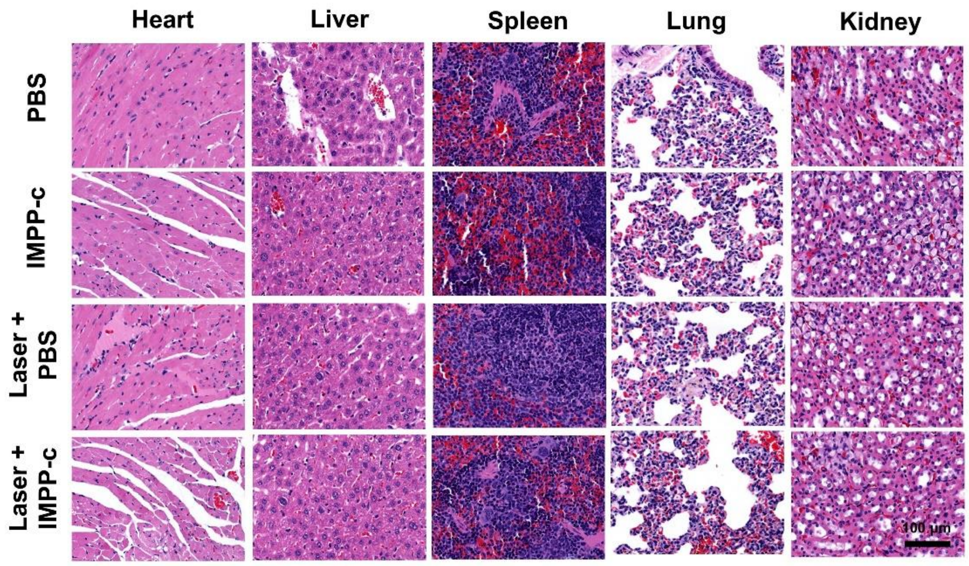

Figure S10. H&E staining of the major organs dissected from treated mice across the four groups.

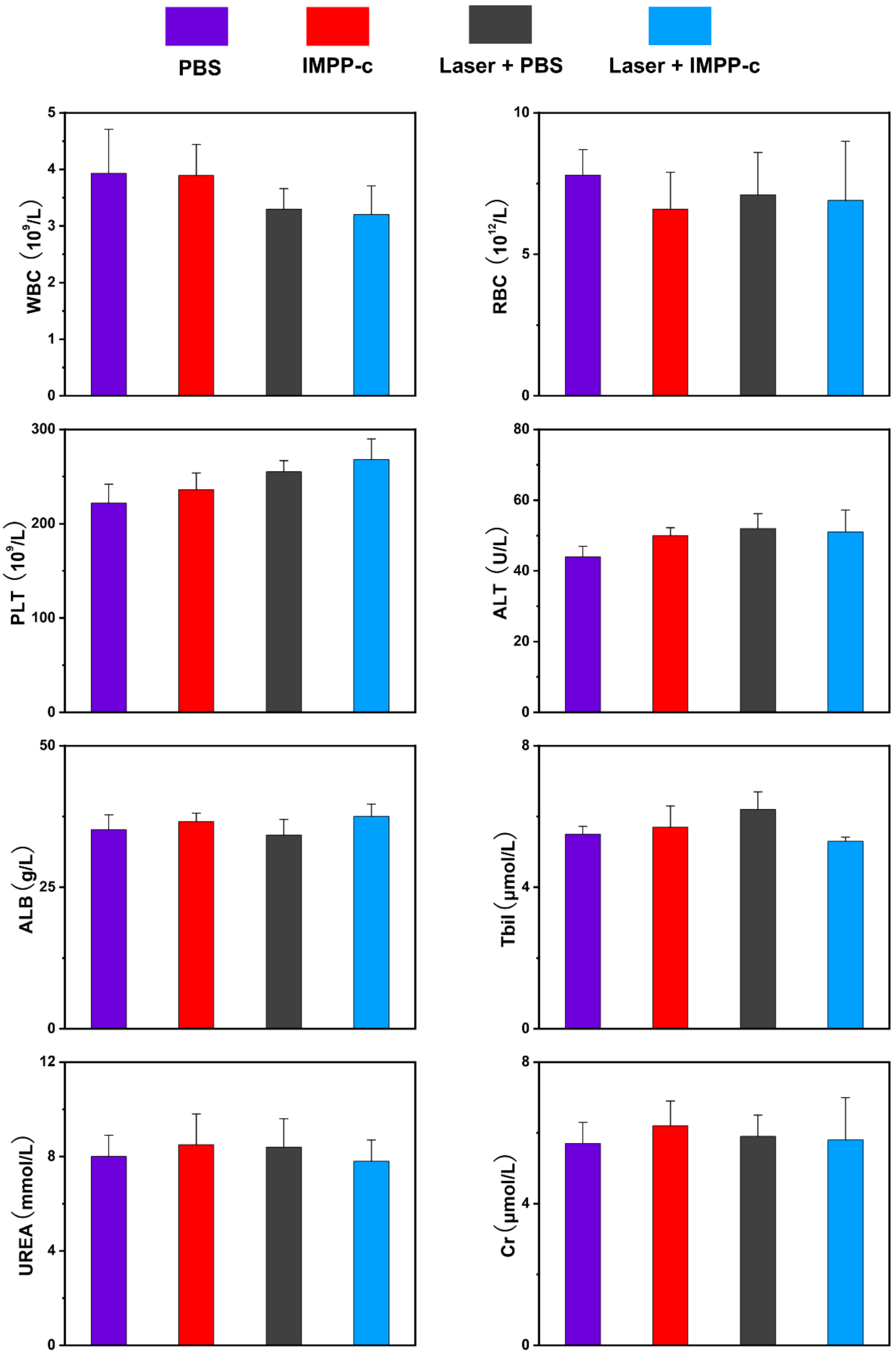

Figure S11. Blood routine and biochemical analyses of nude mice were conducted 15 days post-treatment across all groups (n = 4).
